# Low-Cycle Fatigue Behavior and Microstructural Damage Mechanisms of 316L Austenitic Stainless Steel in Cryogenic Environments

**DOI:** 10.3390/ma19122494

**Published:** 2026-06-10

**Authors:** Sujuan Guo, Guolong Zhang, Junnan Chen, Lei Li, Hui Zhang, Qicong Li, Jian Zhao

**Affiliations:** 1 Key Laboratory of Pressure Systems and Safety, Ministry of Education, School of Mechanical and Power Engineering, East China University of Science and Technology, Shanghai 200237, China; 2 National Key Laboratory of Strength and Structural Integrity, Xi’an 710065, China; 3 Key Laboratory of AI-Aided Airworthiness of Civil Aircraft Structures, School of Aerospace Engineering and Applied Mechanics, Tongji University, Shanghai 201804, China

**Keywords:** cryogenic environment, 316L stainless steel, low-cycle fatigue, microstructural mechanisms, fatigue failure

## Abstract

This study focuses on the low-cycle fatigue behavior and microstructural damage mechanisms of 316L austenitic stainless steel in cryogenic environments to enhance understanding of its fatigue performance and failure mechanisms over a wide temperature range. Uniaxial tensile and strain-controlled low-cycle fatigue tests were performed at 293 K, 173 K, and 77 K; microstructural evolution and damage mechanisms were explored via interrupted tests combined with multiple microscopic techniques and quantitative martensite analysis. The results show that the room temperature fatigue stress response has three stages, while low temperatures induce continuous cyclic hardening that stabilizes quickly; fatigue life increases with lower temperature and strain amplitude, more notably at high strains. Low temperatures enhance strength, increase hardness, slightly reduce plasticity, but maintain good toughness, suppressing crack initiation and propagation with ductile fracture. The findings clarify cryogenic fatigue damage mechanisms, providing experimental and theoretical support for cryogenic pressure-bearing component design and safety assessment.

## 1. Introduction

With the advancement of carbon neutrality goals and the rapid development of the clean energy industry, the production, storage, and utilization of cryogenic liquefied gases such as liquefied natural gas, liquid hydrogen, liquid oxygen, and liquid nitrogen have continued to expand [1,2,3]. As key equipment for the storage and transportation of cryogenic fluids, cryogenic pressure vessels are subjected not only to internal pressure, but also to cyclic loads arising from equipment start-up and shutdown, pressure fluctuations, temperature variations, and transportation vibrations. Fatigue damage has thus become a primary cause of failure for these components [4,5]. Due to its excellent low-temperature toughness, corrosion resistance, and formability, 316L austenitic stainless steel has emerged as the most widely used structural material for cryogenic pressure equipment [6]. Under cryogenic conditions, the mechanical behavior of this steel differs significantly from that at room temperature, making the study of its low-temperature mechanical properties and fatigue performance of both engineering and scientific importance [7,8]. Current research on the mechanical behavior of austenitic stainless steels under cryogenic conditions has focused primarily on uniaxial tensile testing and low-cycle fatigue. For uniaxial tensile studies, systematic experiments have been conducted on S30408, S30403, and S31608 steels in a temperature range of 77–293 K by Chen [9], Huang [10], and Ding [11]; Wang [12] investigated the tensile properties of Fe-Cr-Mn-Mo stainless steel at 293 K and 77 K. Mohsen [13] examined the tensile behavior of AISI 410 stainless steel at room temperature and 77 K. Previous studies indicate that cryogenic conditions can significantly increase the yield and ultimate tensile strengths of austenitic stainless steels, while some alloys exhibited characteristic stages of initial hardening, stress plateau, and secondary hardening in their low-temperature tensile curves. In the field of low-cycle fatigue, Vogt [14,15], Chen [16], and Qing [17] conducted low-cycle fatigue tests on 316L and 316LN stainless steels under both room temperature and cryogenic conditions, revealing the regulatory effect of nitrogen on their low-temperature fatigue performance. Subsequently, Mohsen [18], Toshinori [19], Miao [20], and Xu [21] investigated the low-cycle fatigue behavior of 304, EN1.4301, S30408, and 316LN steels at room temperature and 77 K. These studies demonstrated that low temperatures can markedly enhance the fatigue life of austenitic stainless steels, and that martensitic transformation, dislocation evolution, and deformation twinning during cryogenic fatigue have significant effects on the mechanical response and fatigue life of the austenitic stainless steels. Overall, previous studies have provided a broad exploration of the fatigue behavior and evolution mechanisms of austenitic stainless steels under cryogenic conditions; however, several limitations remain [22]. Existing studies are mostly confined to investigations at room temperature and 77 K, with insufficient attention paid to the transitional mechanisms of cyclic deformation and microstructural evolution at intermediate cryogenic temperatures. Meanwhile, most current research focuses primarily on macroscopic tensile properties and fatigue life, while comparative analysis of the stage-dependent characteristics of cyclic stress response throughout the full fatigue process at different temperatures remains limited. In addition, strain-induced martensitic transformation during cryogenic fatigue has been mostly described qualitatively, lacking quantitative evolution laws of martensite content under the combined effects of temperature, strain amplitude, and cycle number. Furthermore, the essential differences in the dominant microdeformation mechanisms between room temperature and cryogenic conditions have not been systematically clarified.

To address these gaps, this study systematically investigates the uniaxial tensile properties and strain-controlled low-cycle fatigue characteristics of 316L austenitic stainless steel at three representative temperatures: 293 K, 173 K, and 77 K. By comprehensively analyzing the mechanical behavior, cyclic deformation evolution, and temperature-dependent fatigue life, the intrinsic differences in the stage-specific cyclic response features between ambient and cryogenic environments are explicitly elucidated. On this basis, combined with interrupted fatigue tests and multi-scale microstructural characterizations using optical microscopy (OM), scanning electron microscopy (SEM), and transmission electron microscopy (TEM), as well as quantitative measurement of α′-martensite content, the synergistic evolution mechanisms among dislocation substructures, deformation twins, shear bands, and strain-induced martensitic transformation are revealed. This work systematically clarifies the dominant microscopic deformation mechanisms and fatigue damage evolution laws under both ambient and deep cryogenic conditions. These findings not only enrich the baseline database for the cryogenic fatigue performance of 316L stainless steel, but also provide reliable experimental data and theoretical support for the fatigue assessment and safety design of 316L stainless steel used in cryogenic pressure equipment.

## 2. Materials and Methods

### 2.1. Test Materials and Specimens

In this study, 316L austenitic stainless steel was selected as the test material. The steel was produced using a combined electric furnace and argon–oxygen decarburization double-melting process and subsequently solution-treated at 1323 K, followed by water quenching to obtain a uniform and stable austenitic matrix. The main chemical composition of the material is listed in Table 1.

**Figure 1:**
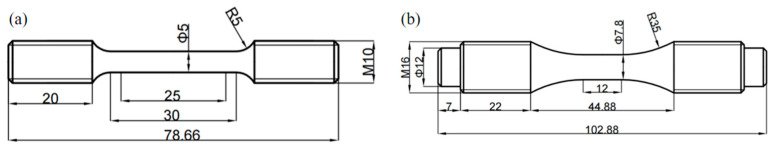
Geometries and dimensions of the specimens: (**a**) static tensile specimen; (**b**) low-cycle fatigue specimen.

To conduct low-temperature mechanical tests, cylindrical rod specimens were designed in accordance with the GB/T 228.3 standard for low-temperature tensile testing and the GB/T 26077 standard for axial strain-controlled fatigue testing [23,24,25]. The tensile specimens had a gauge section diameter of 5 mm, a gauge length of 25 mm, and an overall length of 78.66 mm, while the low-cycle fatigue specimens had a gauge section diameter of 7.8 mm, a gauge length of 12 mm, and an overall length of 102.88 mm. The detailed dimensions of the specimens are shown in Figure 1.

### 2.2. Uniaxial Tensile and Low-Cycle Fatigue Tests in a Cryogenic Environment

**Figure 2:**
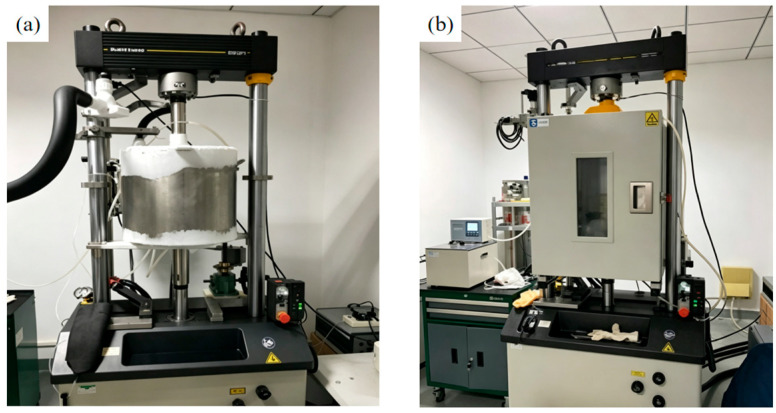
INSTRON fatigue testing system: (**a**) storage tank; (**b**) environmental chamber.

The uniaxial tensile and symmetric strain-controlled low-cycle fatigue tests were systematically conducted to investigate the mechanical properties and fatigue characteristics of 316L austenitic stainless steel under cryogenic conditions. All mechanical tests were performed on an INSTRON 8801 electro-hydraulic servo fatigue testing machine (Instron Corporation, Norwood, MA, USA) equipped with a high-low temperature environmental chamber and a liquid nitrogen forced circulation cooling system (Figure 2), which enabled precise temperature control at 293 K, 173 K, and 77 K. To eliminate thermal gradients within the gauge section and ensure overall thermal equilibrium, the specimens were pre-cooled and held at the target temperature for 35 min prior to loading, ensuring the stability of the testing environment and the reproducibility of the data.

The basic mechanical properties of the material were first obtained through uniaxial tensile testing. Strain was measured using an Epsilon 3542 extensometer (Epsilon Technology Corp, Jackson, WY, USA), and the strain rate was controlled at 0.00025 s^−1^ to eliminate the influence of strain rate sensitivity. Engineering stress–strain curves were collected at different temperatures to determine the yield strength, ultimate tensile strength, and various plasticity indicators, enabling analysis of the effects of temperature on tensile performance. Furthermore, symmetric strain-controlled low-cycle fatigue tests were subsequently conducted with a strain ratio of R = −1. Triangular wave loading was applied at a constant strain rate of 0.004 s^−1^. The tests covered typical strain amplitudes of 0.7%, 0.8%, and 0.9%, with at least three valid repetitions for each condition to ensure statistical reliability. The fatigue life (Nf) was defined as the number of cycles corresponding to a 25% reduction in peak stress relative to the stabilized cyclic stress level during strain-controlled fatigue loading. Real-time monitoring of cyclic stress response, hysteresis loop evolution, and plastic strain energy density was performed to quantitatively reveal the effects of temperature and strain amplitude on the fatigue resistance and cyclic stability of 316L austenitic stainless steel. Prior to fatigue testing, all specimens were mechanically polished to a surface roughness of Ra < 0.2 μm, to minimize the influence of surface machining marks and local surface defects on fatigue crack initiation.

### 2.3. Interrupted Testing and Microstructural Characterization Methods

In order to further study the microstructural evolution mechanisms of 316L austenitic stainless steel’s underlying deformation behavior and fatigue damage in cryogenic environments, interrupted uniaxial tensile and low-cycle fatigue tests were conducted at two representative temperatures: 293 K and 77 K. In the study of microstructural evolution during tensile deformation, strategic sampling was performed based on the characteristic stages of the stress–strain curves, encompassing the as-received material, yield stage, secondary hardening stage, and fracture stage. Corresponding strains of 0%, 10%, 30%, and the fracture state were selected as representative points. These representative strain values enabled the construction of a comprehensive microstructural evolution map covering the entire tensile process. For the evolution of low-cycle fatigue damage, the selection of interruption cycles strictly followed the cyclic stress response characteristic at different temperatures, ensuring that the samples accurately captured the full-life evolution from the initial cycles, through the steady-state stage, to final fracture. Specifically, under 293 K conditions, the selected sampling cycles were 10, 65, 530, and 837 cycles. At 77 K, considering the differences in cyclic hardening behavior compared to room temperature, the sampling points were adjusted to 50, 1700, and 4025 cycles. These sampling points correspond respectively to the initial hardening stage, cyclic stabilization stage, and late failure stage at each temperature, realizing stage-matched microstructural comparison rather than simple fixed-cycle comparison. Systematic observation and analysis of the microstructures at these characteristic stages were conducted to reveal the intrinsic correlation between macroscopic cyclic mechanical responses and microstructural evolution, including dislocation structures and phase transformation behaviors.

Furthermore, this study established a comprehensive characterization framework spanning from macroscopic morphology to microscopic substructure to systematically elucidate the deformation and damage mechanisms of 316L stainless steel at different temperatures. Initially, OM and a ferrite content analyzer were employed to assess the initial microstructure and phase composition of the material. Metallographic specimens were sequentially ground using 800- to 2000-grit sandpapers, mechanically polished, and etched with a nitric acid–glycerol solution (room temperature, 80 s). OM was then used to observe grain morphology, annealing twins, and typical deformation features [26]. To ensure statistical reliability, multi-point random measurements were performed using a FERITSCOPE FMP30 ferrite content analyzer (Helmut Fischer GmbH, Sindelfingen, Germany), and the averaged readings were adopted to evaluate α′-martensite content. According to the empirical conversion relationship reported in the literature [27], the measured magnetic phase content $\omega_{Fe}$ was converted to the mass fraction of α′-martensite $\omega_{\alpha }^{\prime }$ using $\omega_{\alpha }^{\prime }=1.7\omega_{Fe}$, where $\omega_{Fe}$ represents the magnetic phase reading measured by the ferrite analyzer. Prior to martensite measurement, the initial content of residual δ-ferrite in the undeformed base material was tested as the baseline, and the net increase of strain-induced α′-martensite was obtained by deducting the baseline value to exclude the magnetic contribution of inherent δ-ferrite. Subsequently, SEM and a three-dimensional surface profiler were utilized to investigate the fatigue failure mechanisms. Fractured specimens were ultrasonically cleaned in anhydrous ethanol, and SEM was used to conduct detailed morphological characterization of crack initiation regions, steady-state propagation regions, and instant fracture zones, allowing analysis of crack nucleation and growth behavior [28].

In addition, the height distribution profiles of the fracture surfaces were extracted using a three-dimensional surface profiler [29], providing insights into the influence of temperature on crack dynamics and fracture modes from both geometric and microstructural perspectives. Finally, TEM was employed to characterize the morphological evolution of dislocation substructures and reveal microstructural mechanisms of deformation strengthening at cryogenic temperatures. To ensure the accuracy of substructure characterization, TEM specimens were mechanically thinned to 50 μm and subsequently prepared using a double-jet electrochemical thinning method in a methanol–perchloric acid electrolyte solution at −30 °C. Key features, including dislocation configurations, shear bands, stacking faults, and deformation twins, were clearly captured by TEM [30,31].

## 3. Cyclic Deformation Behavior and Fatigue Life Characteristics

### 3.1. Uniaxial Tensile Behavior of 316L Stainless Steel at Cryogenic Temperatures

**Figure 3:**
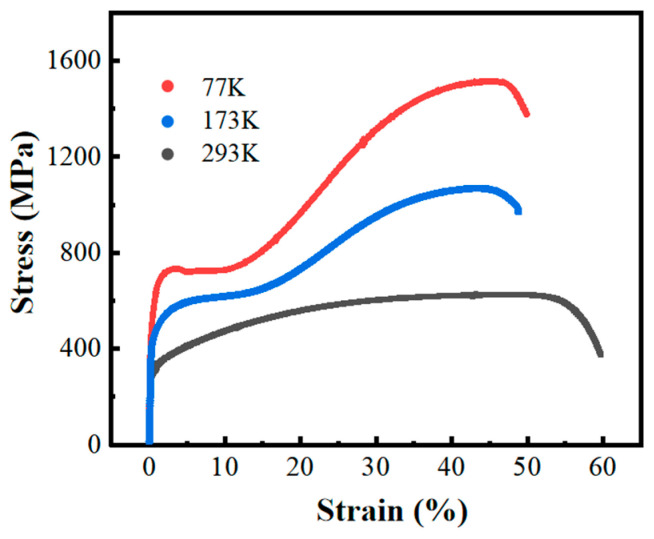
Tensile stress–strain curves of the material at various temperatures.

This study first investigated the tensile deformation behavior of 316L austenitic stainless steel at different temperatures. As shown in Figure 3, at room temperature (293 K), the stress increased gradually and continuously with strain, exhibiting characteristic parabolic hardening behavior. In contrast, under cryogenic conditions of 173 K and 77 K, the tensile curves developed in a stepped manner, accompanied by the emergence of a pronounced yield plateau. With decreasing temperature, the yield plateau shortened, while the secondary hardening rate increased. Previous studies have indicated [10] that the formation of the yield plateau may originate from the evolution of dislocation behavior at low temperatures, where local adiabatic heating induces dislocation annihilation and strain-driven dislocation multiplication, reaching a dynamic equilibrium. At the same time, stress softening associated with deformation-induced martensitic transformation and the pinning effect of newly formed phases collectively govern the evolution of the yield stage.

**Figure 4:**
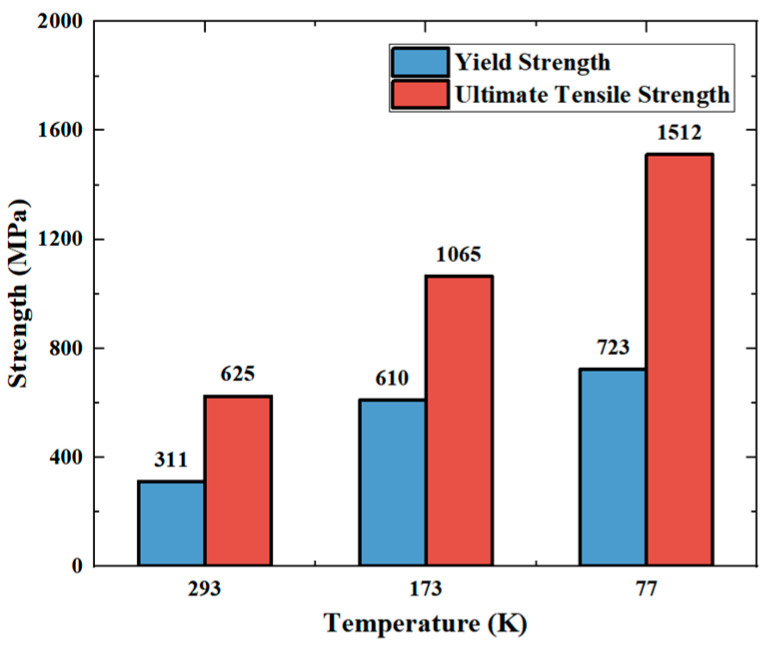
Material strength at various temperatures.

Furthermore, strength parameters of 316L stainless steel across the 293 K to 77 K range were quantitatively analyzed, as shown in Figure 4. The results indicate that both the yield strength and tensile strength of the material rise obviously with decreasing temperature, with distinct differences in strength increments across different temperature ranges. As the temperature decreases from 293 K to 173 K, the yield strength increases by 96.1%, while a further reduction to 77 K only leads to an increment of 18.5%. Correspondingly, the tensile strength increases by 70.4% from 293 K to 173 K and by 42.0% from 173 K to 77 K. Relative to 293 K, the total increment of tensile strength at 77 K reaches approximately 141.9%, slightly higher than the 132.5% increment in yield strength, which demonstrates that low temperature imposes a more marked strengthening effect on tensile strength than on yield strength. The observed cryogenic strengthening effect can be attributed to multiple synergistic mechanisms, including second-phase strengthening from deformation-induced martensitic transformation, dislocation loop formation due to the suppression of equilibrium vacancy concentration at low temperatures [32], and increased resistance to dislocation motion caused by dislocation pile-up at grain boundaries coupled with inhibited thermal activation [33,34].

**Figure 5:**
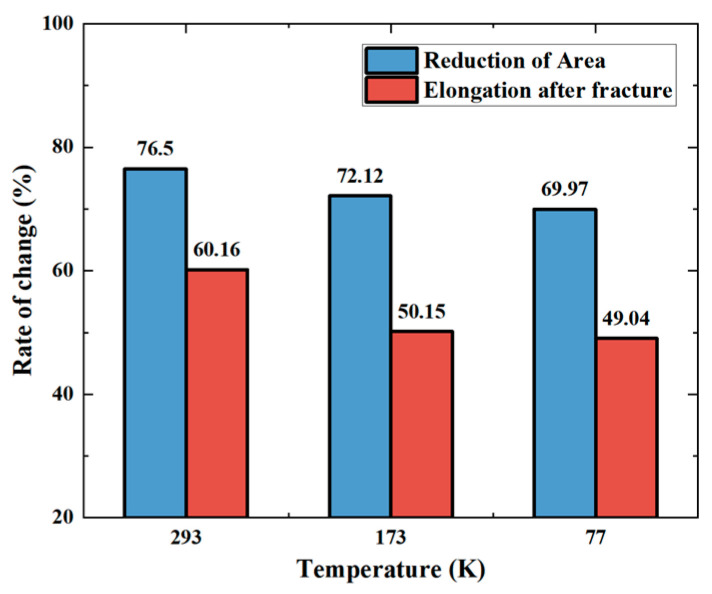
Reduction of area and elongation after fracture of the material at various temperatures.

Finally, the plastic deformation capability of the material was assessed through reduction of area and elongation after fracture, as shown in Figure 5. The results indicate that, although plasticity slightly decreased with decreasing temperature, the overall reduction was limited, suggesting that the material maintains good ductility even at cryogenic temperatures, satisfying the requirements for low-temperature containers. The slight decrease in plasticity reflects strain mismatch caused by the high-hardness martensitic phase; however, mechanically induced twinning formed during deformation effectively may increase the number of active slip systems and promote more uniform strain distribution, thereby compensating for the loss of ductility. The synergistic combination of hardening and toughening may be the reason for ensuring the excellent strength reserve and deformation stability of 316L stainless steel under extreme low temperature conditions [35,36].

### 3.2. Cyclic Deformation Behavior of 316L Stainless Steel Under Cryogenic Conditions

This section presents a systematic investigation of the cyclic deformation behavior of 316L austenitic stainless steel under cryogenic conditions. Low-cycle fatigue tests were conducted at various temperatures and strain amplitudes to systematically examine the material’s cyclic stress response, hysteresis loop evolution, plastic strain amplitude evolution, and the applicability of the Masing criterion. The study aims to reveal, from both macroscopic mechanical responses and underlying microstructural mechanisms, the regulatory effects of temperature on cyclic strengthening pathways and damage accumulation processes.

**Figure 6:**
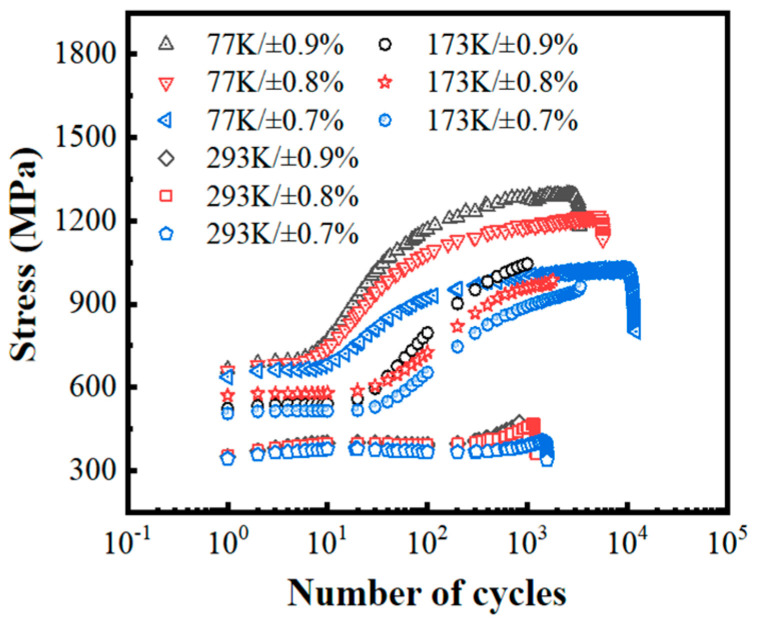
Cyclic stress amplitude response curves at various temperatures.

Figure 6 presents the cyclic stress amplitude evolution curves of 316L stainless steel at different temperatures and strain amplitudes. At 293 K, the stress amplitude exhibits a three-stage behavior: initial hardening, subsequent softening, and secondary hardening prior to fracture. In contrast, under cryogenic conditions of 173 K and 77 K, the material enters a rapid hardening stage shortly after a brief initial slow hardening period, eventually reaching cyclic saturation until fracture. The analysis suggests that the cyclic stress level is modulated by the coupled effects of temperature and applied strain amplitude. Notably, after 10 cycles, low temperatures significantly enhance interactions between dislocations and newly formed phases, leading to increasingly pronounced stress differences corresponding to the same strain amplitude as temperature decreases.

**Figure 7:**
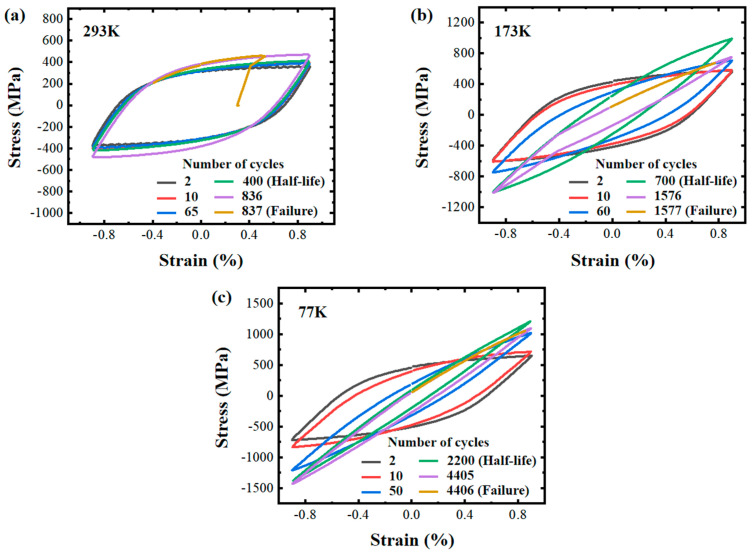
Hysteresis loops at various temperatures: (**a**) 293 K; (**b**) 173 K; (**c**) 77 K.

To examine the evolution of hysteresis behavior, Figure 7 presents the hysteresis loops at a strain amplitude of 0.9% under different temperatures. At 293 K, the increase in hysteresis loop area and stress amplitude is relatively small, indicating stable plastic dissipation and damage accumulation per cycle. When the temperature is decreased to 173 K or 77 K, the maximum stress rises sharply with cycle number, significantly enhancing cyclic hardening. In the later stage of cycling, the reduction in hysteresis loop area, loss of symmetry, and the appearance of inflection points during unloading are primarily attributed to the nucleation of microcracks, which reduce the effective load-bearing area and induce stress concentration effects [37].

**Figure 8:**
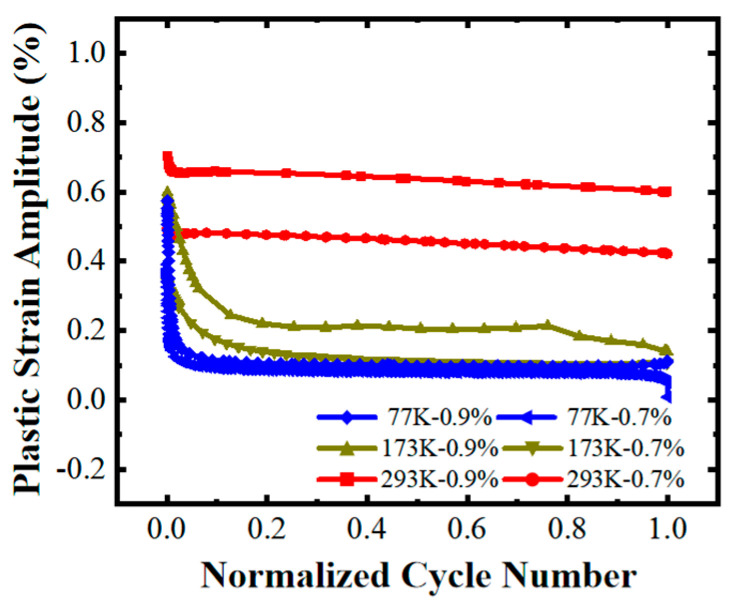
Evolution of cyclic plastic strain amplitude versus normalized number of cycles under various applied strain amplitudes at different temperatures.

Furthermore, the evolution of cyclic plastic strain amplitude under different conditions was systematically analyzed, as shown in Figure 8. The results indicate that, under all conditions, the plastic strain amplitude rapidly decreases over the initial cycles and eventually stabilizes. Cryogenic environments strongly suppress the material’s plastic deformation capability, resulting in much lower stabilized plastic strain levels than at room temperature, and the differences between various strain amplitudes become less pronounced at lower temperatures. This behavior may be attributed to the coupled effects of low temperature and cyclic deformation, which facilitate the extensive formation of deformation-induced martensite [38]. The newly generated strengthened martensitic phase can hinder dislocation motion, thereby reducing and homogenizing the plastic deformation capacity under cyclic loading [39].

**Figure 9:**
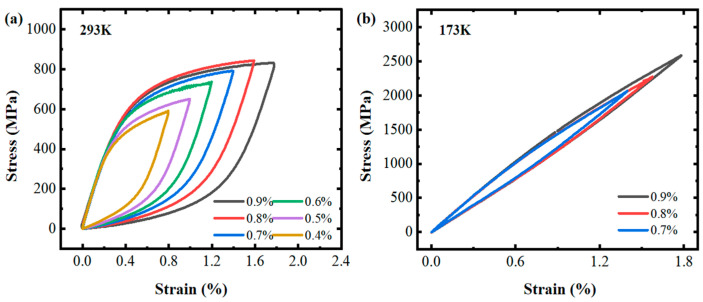
Hysteresis loops under various applied strain amplitudes at (**a**) 293 K and (**b**) 77 K.

Finally, the Masing characteristics of 316L stainless steel were evaluated using the stabilized hysteresis loops at half-life cycles under different strain amplitudes, as shown in Figure 9. To facilitate comparison, the lower endpoints of the hysteresis loops were translated to the coordinate origin for normalization. According to the classical phenomenological Masing criterion, materials exhibiting Masing behavior should show good coincidence of the elastic and yielding segments under different strain amplitudes. It can be observed that, with increasing strain amplitude, the yield segments of the hysteresis loops at all temperatures exhibit relatively poor coincidence, while the size of the yield surface expands progressively during cyclic deformation, indicating a non-Masing-type cyclic response characteristic. Previous studies have suggested that non-Masing-type behavior is closely associated with the instability of internal phase evolution and dislocation substructure during cyclic deformation [40]. Combined with the subsequent microstructural observations in the present study, including deformation-induced martensitic transformation and dislocation structure evolution, these factors are considered to be closely related to the observed hysteresis response characteristics of 316L stainless steel under cryogenic fatigue conditions.

### 3.3. Fatigue Life Distribution of 316L Stainless Steel Under Cryogenic Conditions

**Figure 10:**
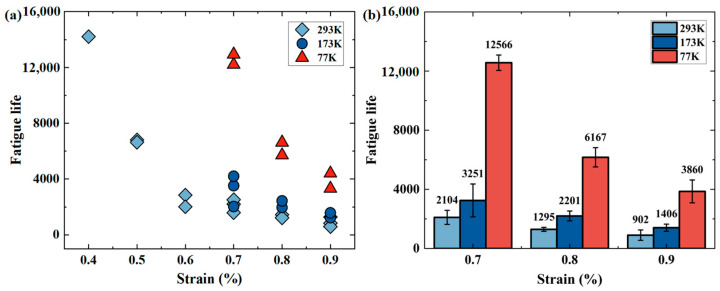
Low-cycle fatigue behavior of 316L stainless steel at different temperatures: (**a**) fatigue life at various temperatures and strain amplitudes; (**b**) mean fatigue life statistics at various temperatures and strain amplitudes.

This section evaluates the low-cycle fatigue behavior of 316L stainless steel over a temperature range of 77–293 K and strain amplitudes from 0.7% to 0.9%, aiming to clarify the coupled effects of temperature and strain amplitude on the material’s fatigue failure pathways. A series of fatigue tests were conducted under the above working conditions at different strain energy levels to obtain life response data under various conditions, with the evolution trends and comprehensive statistical results shown in Figure 10.

To elucidate the intrinsic relationship among strain level, environmental temperature, and fatigue life, the fatigue life response characteristics of the material under different temperatures and strain amplitudes were systematically analyzed, as illustrated in Figure 10a. The results indicate that, at a given strain amplitude, reducing the environmental temperature markedly enhances the fatigue resistance of the material and retards the damage accumulation process, thereby leading to a substantial improvement in fatigue life. At a constant temperature, the fatigue life exhibits a nonlinear decreasing trend with increasing total strain amplitude. Since plastic deformation damage becomes dominant at higher strain levels, the rate of fatigue life reduction gradually decreases with increasing strain amplitude, exhibiting a typical low-cycle fatigue evolution behavior.

To further quantify the coupled effects of temperature and strain amplitude on low-cycle fatigue life, the average fatigue lives under different testing conditions were statistically analyzed, as shown in Figure 10b. The error bars represent ±1 standard deviation of the samples, reflecting the scatter of the experimental data. Comparative analysis indicates that the fatigue response of the material to low-temperature environments exhibits pronounced strain dependence. Under low strain amplitude loading conditions, reducing the temperature produces a particularly significant enhancement in fatigue life. However, as the strain amplitude increases, this beneficial effect of low temperature gradually diminishes. This behavior reveals the synergistic regulation mechanism of temperature and strain amplitude on fatigue damage evolution, indicating that the improvement in fatigue life induced by low temperature is strongly coupled with the strain level under low-cycle fatigue conditions.

To further support engineering fatigue design and life evaluation, the classical Manson–Coffin–Basquin (MCB) relationship was adopted to establish the low-cycle fatigue life prediction model. The total strain amplitude can be decomposed into elastic and plastic strain components, following the unified MCB equation:

(1) $$\frac{∆\varepsilon }{2}=\frac{\sigma_{f}^{\prime }}{E}{\left(2N_{f}\right)}^{b}+\varepsilon_{f}^{\prime }{\left(2N_{f}\right)}^{c}$$

where ∆ε/2 is the total strain amplitude, $N_{f}$ is the fatigue failure cycle number, E is the elastic modulus, $\sigma_{f}^{\prime }$ and b represent the fatigue strength coefficient and fatigue strength exponent corresponding to the Basquin elastic strain term, and $\varepsilon_{f}^{\prime }$ and c are the fatigue ductility coefficient and fatigue ductility exponent for the Coffin–Manson plastic strain term.

**Figure 11:**
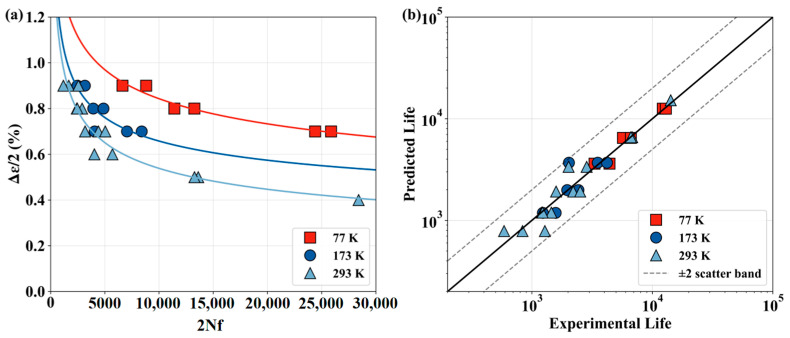
Manson–Coffin–Basquin model fitting and life prediction verification: (**a**) strain–life fitting curves at different temperatures; (**b**) comparison between experimental fatigue life and model predicted life.

Based on the experimental data at 293 K, 173 K and 77 K, the MCB model was fitted, and the corresponding strain–life fitting curves are shown in Figure 11a. The predicted fatigue life calculated from the fitted Manson–Coffin–Basquin equation is compared with the experimental data in Figure 11b, which verifies that the established life prediction model possesses good accuracy and reliability. The complete fitted MCB parameters at different temperatures are summarized in Table 2 and can provide theoretical basis and reference data for engineering fatigue analysis, structural design, and safety assessment of 316L stainless steel components under cryogenic service conditions.

## 4. Microstructural Failure Mechanisms of 316L Stainless Steel

### 4.1. Microstructural Observation and Mechanism Analysis During Tensile Deformation

**Figure 12:**
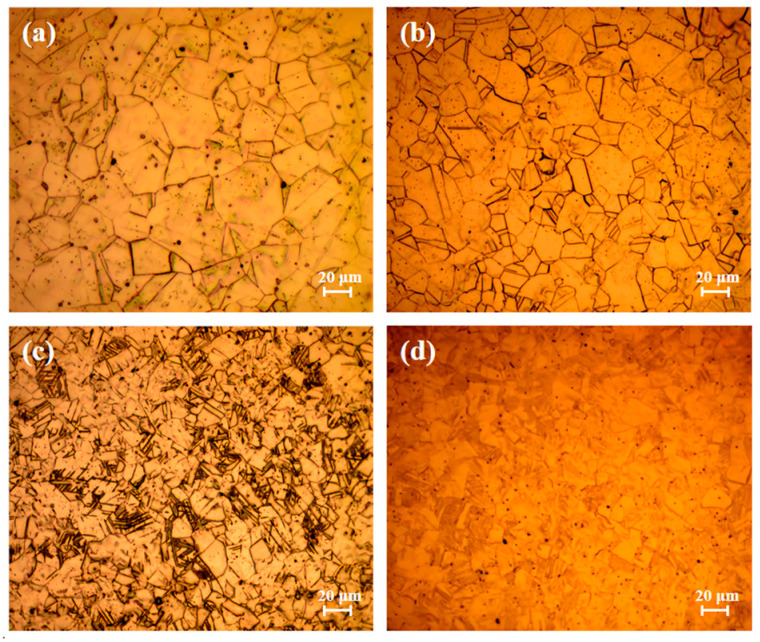
Metallographic microstructures of interrupted tensile specimens at 293 K: (**a**) as-received metal, (**b**) 10% strain, (**c**) 30% strain, (**d**) fracture.

**Figure 13:**
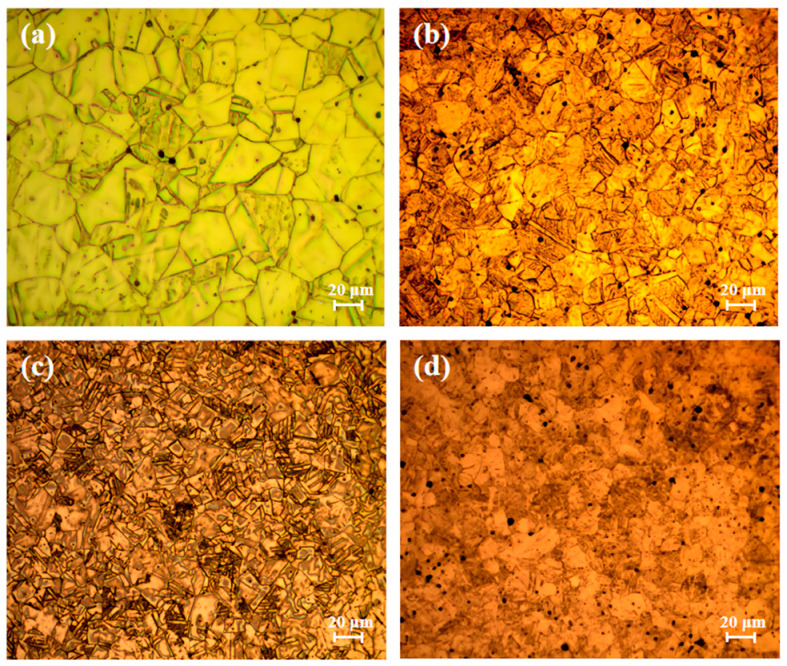
Metallographic microstructures of interrupted tensile specimens at 77 K: (**a**) as-received metal, (**b**) 10% strain, (**c**) 30% strain, (**d**) fracture.

In this section, the microstructural evolution of 316L stainless steel at different tensile stages (strain levels of 0%, 10%, 30%, and fracture) was systematically examined using OM at 500× magnification under 293 K and 77 K conditions, aiming to reveal the effects of temperature and deformation on microstructural development and deformation mechanisms. The characterization results are shown in Figure 12 and Figure 13.

At 293 K, the as-received 316L stainless steel (Figure 12a) primarily consists of equiaxed, polygonal austenite, with a minor presence of annealing twins and residual elongated δ-ferrite. Although ferrite exhibits good ductility and toughness, its relatively low strength and hardness can adversely affect the overall material performance, for example by reducing corrosion resistance and increasing the tendency for cracking during thermal processing. At 10% tensile strain (Figure 12b), mechanical twins nucleate at grain boundaries and propagate into the grains under localized stress concentrations. The formation of mechanical twins consumes part of the strain energy, thereby effectively reducing local stress levels [41]. As the strain increases to 30% and fracture (Figure 12c,d), a substantial amount of fine, lath-like α′-martensite forms within the material, with its fraction clearly increasing with the degree of deformation.

Under the cryogenic condition of 77 K, the microstructural evolution differs from that observed at room temperature. The as-received microstructure at 77 K (Figure 13a) presents similar overall morphological features to that at 293 K. Compared to room temperature, at 10% tensile strain (Figure 13b), a large volume of martensitic structure rapidly develops within 316L stainless steel, indicating that cryogenic conditions significantly promote deformation-induced martensitic transformation. With further strain, the martensite fraction continues to increase (Figure 13c,d). In addition, because the stacking fault energy decreases at low temperatures, the number of mechanical twins formed at 77 K is notably higher than formed at 293 K. Twinning serves as a homogeneous shear mechanism that facilitates more uniform plastic deformation. Moreover, the presence of numerous parallel twin boundaries effectively subdivides and refines austenite grains, significantly enhancing the material’s strength at cryogenic temperatures.

**Figure 14:**
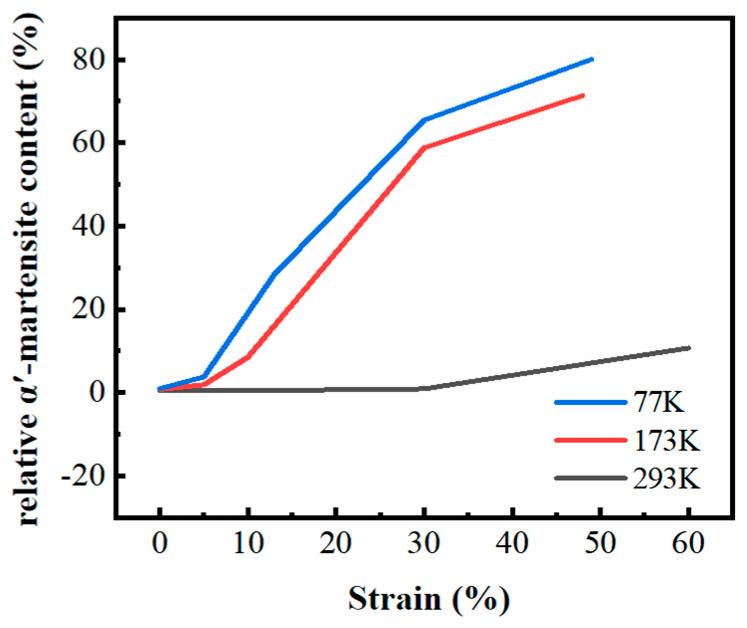
Evolution of α′-martensite content as a function of tensile strain for 316L stainless steel at various temperatures.

The above microstructural observations indicate that temperature and strain conditions strongly influence martensite formation and twinning activity. Based on these observations, the evolution of α′-martensite fraction with strain was further analyzed, as shown in Figure 14. Martensitic transformation is primarily driven by the combined effects of temperature and deformation [42].

From a thermodynamic perspective, when the environmental temperature is lower than the martensite start temperature $M_{s}$, the Gibbs free energy difference between the austenite and martensite phases provides the intrinsic driving force for phase transformation. A lower temperature leads to a greater degree of undercooling and a stronger tendency for spontaneous martensitic transformation.

In this study, $M_{s}$ was estimated using the widely adopted empirical composition–temperature relationship [43]:

(2) $$M_{s}=731-227(C+N)-17.6\mathrm{Ni}-22.5\mathrm{Mn}-17.3\mathrm{Cr}-16.2\mathrm{Mo}$$

where C, N, Ni, Mn, Cr, and Mo represent the mass percentages (wt.%) of the corresponding alloying elements in 316L stainless steel. The calculated $M_{s}$ value for the present alloy is 175.44 K.

When the test temperature lies between $M_{s}$ and the maximum temperature for deformation-induced martensitic transformation $M_{d}$, thermodynamic driving force alone is insufficient to sustain extensive martensitic transformation, and external mechanical deformation is required to promote the phase transition [44]. When the temperature exceeds $M_{d}$, it becomes difficult for deformation-induced martensitic transformation to occur in the present 316L stainless steel.

As shown in Figure 14, at 293 K, the phase transformation in 316L stainless steel proceeds relatively slowly. When the tensile strain is below 30%, the α′-martensite fraction remains at a very low level and only increases sharply after exceeding this critical strain, continuing up to specimen fracture. Concurrently, the formation of the intermediate ε-martensite is limited and exhibits a gradual change. Under cryogenic conditions (173 K and 77 K), the evolution of α′-martensite exhibits a markedly different trend: its initial growth is slow (strain < 5%), followed by a rapid proliferation stage, and the growth rate gradually diminishes beyond 30% strain.

The temperature dependence and cumulative extent of martensitic transformation exert a dominant effect on the macroscopic mechanical properties of 316L stainless steel. Combined with the martensite evolution characteristics in Figure 14, the differences in tensile deformation behavior and strength performance at various temperatures can be well interpreted. In terms of tensile stress–strain responses (Figure 3), the slow increase in flow stress at the early tensile stage (strain < 30%) is mainly governed by dislocation accumulation and minor ε-martensite formation. At the middle and late deformation stages, the extensive nucleation of high-hardness α′-martensite remarkably raises the work-hardening rate and dominates the rapid stress rise. At cryogenic temperatures, early martensite formation corresponds to the yield plateau in stress–strain curves within 5–10% strain, while continuous martensite proliferation at higher strains induces secondary hardening. Overall, martensitic transformation coupled with dislocation accumulation serves as the core mechanism controlling the work-hardening behavior under different thermal conditions. From the perspective of strength variation with temperature (Figure 4), the gradual temperature reduction from 293 K to 173 K promotes substantial generation of α′-martensite, which effectively improves the yield strength and ultimate tensile strength of the material. As the temperature further decreases to 77 K, the phase transformation driving force gradually reaches saturation, resulting in limited additional martensite increment and a weakened strength enhancement effect. Meanwhile, the abundant high-hardness α′-martensite strengthens the matrix considerably but also restricts the plastic coordination ability of the material. It is demonstrated that temperature and strain jointly regulate the nucleation and development of deformation-induced martensite, thereby determining the macroscopic mechanical performance of 316L stainless steel.

**Figure 15:**
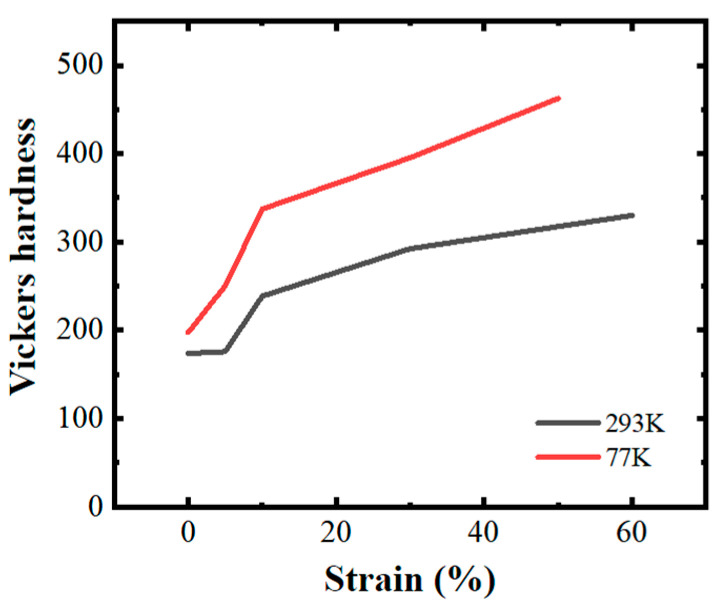
Hardness evolution of the material during tensile deformation.

To further quantify the contribution of microstructural evolution to strain hardening, hardness measurements were performed on specimens at different tensile stages (Figure 15). The results show that the material hardness under cryogenic conditions is generally higher than at room temperature and increases with strain. At low strain levels, hardness rises rapidly, mainly due to the rapid formation of martensite and the accumulation of dislocations. At high strain levels, although martensite formation slows and dislocation density approaches saturation, the hardness remains relatively high. These results indicate that martensitic evolution and dislocation accumulation play a critical role in strain hardening under varying temperature and strain conditions and correlate well with observed changes in macroscopic mechanical behavior.

### 4.2. Fractographic Analysis of Fatigue Fractures

In this section, fatigue specimens of 316L stainless steel tested at a strain amplitude of 0.9% and at three representative temperatures (293 K, 173 K, and 77 K) were systematically examined. SEM and a three-dimensional (3D) profilometer were employed to characterize fracture surfaces, aiming to elucidate the influence of temperature on fatigue fracture behavior. Prior to testing, specimen sections located at least 3 mm away from the fracture surface were carefully prepared, ultrasonically cleaned in anhydrous ethanol, and dried to remove surface contaminants and oils, ensuring high-precision observations. Three-dimensional profilometry scans were performed along the radial path from crack initiation through steady-state propagation to final fracture. By combining fracture height maps with cross-sectional profiles, both qualitative and quantitative analyses of fracture surface features at different temperatures were achieved.

**Figure 16:**
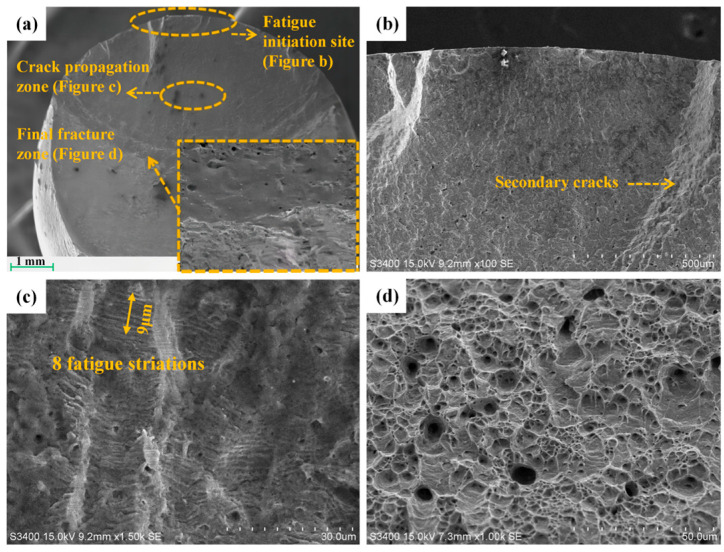
Fatigue fracture morphologies of 316L stainless steel at a strain amplitude of 0.9% at 293 K: (**a**) macro-morphology, (**b**) crack initiation site, (**c**) propagation region, (**d**) final fracture zone.

**Figure 17:**
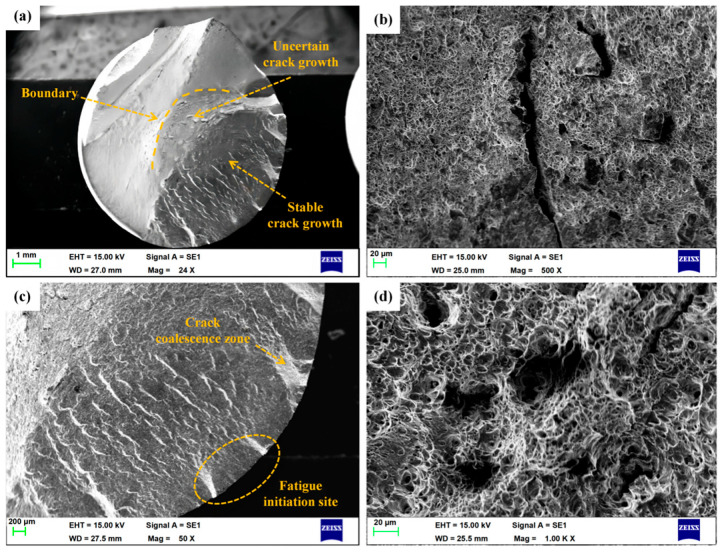
Fatigue fracture morphologies of 316L stainless steel at a strain amplitude of 0.9% at 173 K: (**a**) macro-morphology, (**b**) unstable crack growth region, (**c**) crack initiation site, (**d**) final fracture zone.

**Figure 18:**
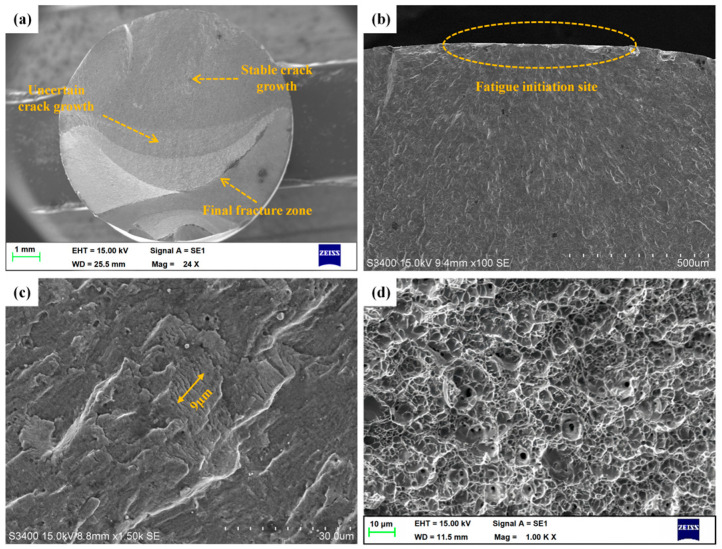
Fatigue fracture morphologies of 316L stainless steel at a strain amplitude of 0.9% at 77 K: (**a**) macro-morphology, (**b**) crack initiation site, (**c**) fatigue striations, (**d**) final fracture zone.

Fatigue fracture surfaces of 316L stainless steel were characterized by SEM at a strain amplitude of 0.9% at temperatures of 293 K, 173 K and 77 K, and the corresponding results are presented in Figure 16a–d, Figure 17a–d, and Figure 18a–d, respectively. Figure 16a–d display the overall and local micro-morphologies of fatigue fracture at 293 K. As shown in Figure 16a, the fracture surface consists of a crack initiation zone, a crack propagation zone, and a final fracture zone, exhibiting a multi-source crack initiation characteristic. Cracks initiate from the specimen surface and propagate inward. Fan-shaped river-like patterns can be observed in Figure 16b, which serve as an important morphological feature for identifying crack initiation sites. Secondary cracks in this view form due to the elevated stress at the crack tip when the main crack propagation is blocked, inducing lateral crack extension. A smooth boundary between the propagation zone and final fracture zone can be clearly distinguished, with a remarkable difference in surface roughness. The propagation zone exhibits a flat morphology owing to the low crack growth rate, while the final fracture zone shows a rough surface resulting from rapid unstable crack propagation. In Figure 16c, fatigue striations present an irregular parallel wave pattern perpendicular to the crack propagation direction, with an average width of approximately 1.125 μm. A large number of dimples and microvoids are distributed in the final fracture zone in Figure 16d, indicating that the fatigue failure of 316L stainless steel at 293 K is dominated by the ductile fracture mode.

Figure 17a–d show the fatigue fracture micro-morphology at 173 K and a strain amplitude of 0.9%. The crack propagation zone in Figure 17a can be divided into a stable propagation zone and an unstable propagation zone with a clear boundary. The stable propagation zone possesses a flat fracture surface, which is attributed to the low crack growth rate and repeated cyclic loading. During the unstable propagation stage, the crack growth rate increases sharply, leading to rapid tearing of the specimen and the formation of typical ductile dimple features, as illustrated in Figure 17b. All fatigue cracks in Figure 17c originate from the specimen surface. Surface scratches and machining defects easily induce local stress concentrations, and non-uniform grain contraction at low temperature further aggravates stress concentration and promotes crack initiation. Obvious crack coalescence can be found within the initiation zone. Cracks preferentially propagate along crystallographic planes and orientations with lower resistance, and the intersection of cracks with different propagation paths forms a micro-morphology with distinct height undulations. Compared with the fracture morphology at 293 K, tearing ridges become more prominent at 173 K. Although the fracture still maintains ductile characteristics, the overall dimple morphology reflects a certain degradation in plasticity (Figure 17d).

Figure 18a–d present the fatigue fracture micro-morphology at 77 K and a strain amplitude of 0.9%. Compared with the fracture morphology at 173 K, fewer observable crack initiation sites can be observed in Figure 18a, indicating a reduced number of active crack sources under cryogenic conditions. The flatness of the stable propagation zone in Figure 18b is further improved. Low temperature reduces the crack growth rate and weakens the plastic deformation capacity of the material, and the fracture surface becomes much smoother under repeated cyclic loading. The width of fatigue striations in Figure 18c is approximately 1 μm, which is smaller than that at 293 K. Fatigue striation width can qualitatively reflect the relative crack growth rate. Generally, wider striations tend to imply a faster local crack propagation rate. On this basis, the broader fatigue striations observed at 293 K suggest a relatively higher crack growth rate compared with 77 K, which reasonably explains the shorter fatigue life at room temperature. The size and depth of dimples in the final fracture zone (Figure 18d) are obviously lower than those at 293 K and 173 K. Dimple morphology is jointly controlled by material plasticity and internal inclusions. Under the cryogenic condition of 77 K, the increasing fraction of deformation-induced martensitic phases not only enhances cyclic hardening behavior, but also introduces local deformation incompatibility and stress concentration at martensite/austenite interfaces, thereby further weakening the local plastic deformation capability and resulting in finer and shallower dimples in the final fracture region.

**Figure 19:**
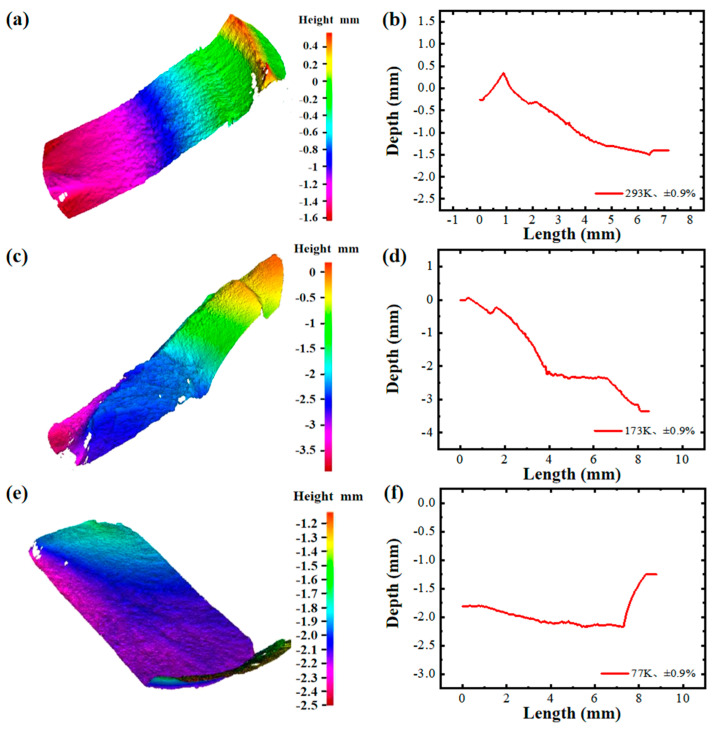
Fatigue fracture characteristics of 316L stainless steel at a strain amplitude of 0.9% at different temperatures: (**a**) 3D morphology of the fracture surface, (**b**) height variation profile at 293 K; (**c**) 3D morphology of the fracture surface, (**d**) height variation profile at 173 K; (**e**) 3D morphology of the fracture surface, (**f**) height variation profile at 77 K.

On the basis of SEM micro-morphology analysis, a 3D profilometer was used to quantitatively characterize the elevation features of fatigue fracture surfaces at the three temperatures, as shown in Figure 19. The evolutionary law of 3D elevation is strongly consistent with the microstructural characteristics of fracture surfaces. The 3D cloud map and radial height curve of the fracture surface at 293 K show that the elevation increases gently from the crack initiation zone to the final fracture zone, with a maximum height difference of approximately 1.75 mm. The overall fracture undulation remains relatively uniform without abrupt local fluctuations, which is consistent with the good plastic deformation capability and relatively stable crack propagation behavior of the material at room temperature. At 173 K, the overall undulation amplitude of the fracture surface increases significantly, and the maximum elevation difference reaches 3.25 mm, which is much higher than that at 293 K. This pronounced roughness is mainly associated with the simultaneous initiation, propagation, and coalescence of multiple fatigue cracks, leading to a more tortuous propagation path and substantial local height fluctuations on the fracture surface. In addition, the reduced plasticity under low-temperature conditions promotes unstable local fracture behavior during the late fatigue stage, further increasing the fracture surface irregularity. In contrast, the fracture surface at 77 K exhibits the flattest and most regular morphology, with a total elevation difference of only about 1 mm. The height varies relatively smoothly from the crack initiation region to the stable propagation region, followed by a sharp increase in the final fracture zone. Although the ductility of 316L stainless steel is further reduced under the cryogenic condition of 77 K, the fracture process appears to be dominated by fewer principal crack propagation paths with reduced multi-crack interaction and secondary crack coalescence. Consequently, the fracture morphology becomes more localized and directional, resulting in a relatively smoother overall fracture surface despite the reduced plastic deformation capability.

### 4.3. Microstructural Evolution and Mechanistic Analysis During Fatigue

To clarify the regulation mechanism of cryogenic environment on the microstructural evolution and cyclic stress response of 316L stainless steel during low-cycle fatigue, fatigue deformation behavior at 77 K cryogenic temperature was systematically investigated with 293 K (room temperature) as the reference. TEM was employed to characterize the microstructures of specimens at different fatigue stages and fracture states. Combined with the quantitative measurement of α′-martensite content, the evolution of dislocation substructures, the initiation of shear bands and twins, and the law of deformation-induced phase transformation at the two temperatures were comparatively analyzed, thereby establishing the intrinsic correlation between microstructural evolution and macroscopic fatigue response.

**Figure 20:**
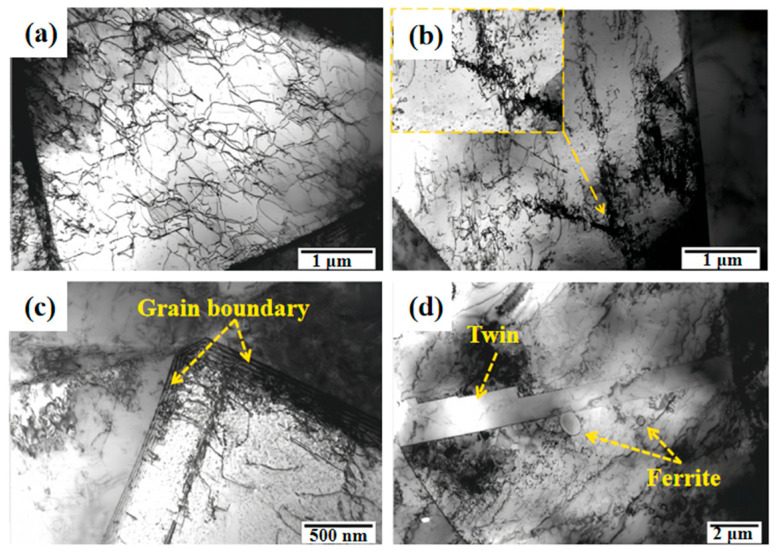
TEM micrographs showing the microstructural evolution of 316L stainless steel at 293 K and a strain amplitude of 0.9% at 10 cycles: (**a**) dislocation distribution, (**b**) dislocation veins, (**c**) dislocation piling-up, (**d**) twins and ferrite grains.

**Figure 21:**
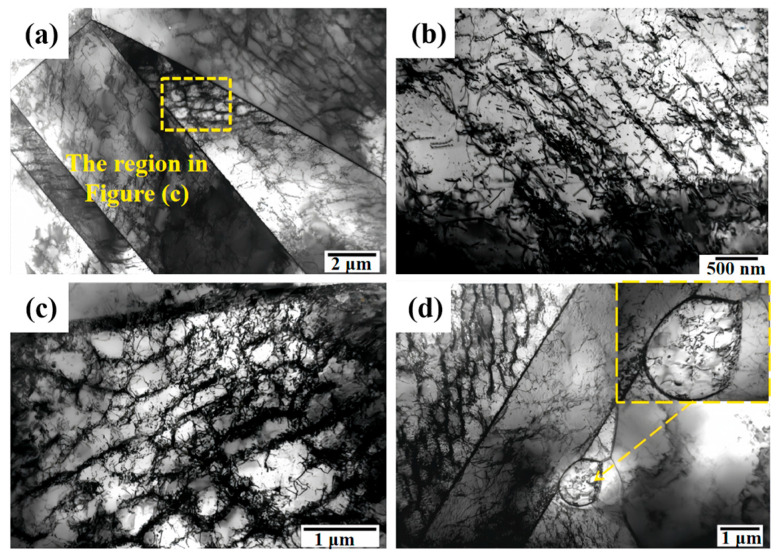
TEM micrographs showing the microstructural evolution of 316L stainless steel at 293 K and a strain amplitude of 0.9% at 65 cycles: (**a**) dislocation morphology, (**b**) dislocation veins, (**c**) magnified view of dislocation walls, (**d**) twins and ferrite grains.

316L stainless steel exhibits staged microstructural evolution at different cycle numbers at 293 K. The microstructure at the initial cyclic hardening stage (10 cycles) is presented in Figure 20a–d. A large number of planar dislocations rapidly nucleate in the matrix induced by high strain amplitude, which are mainly characterized by discrete dislocation lines, network configurations, and slight local accumulation. Dense dislocation veins form in stress-concentrated regions, and dislocation slip is constrained by grain boundaries, resulting in obvious dislocation pile-up, indicating that grain boundaries can effectively hinder dislocation motion and propagation. The matrix is dominated by austenite, accompanied by ferrite phases with smooth grain boundaries and no internal twins, as well as annealing twins with straight grain boundaries and thick lamellae. At the cyclic softening stage (65 cycles), the corresponding microstructural evolution is shown in Figure 21a–d. With the increase in cycle number, dislocation density rises continuously and gradually develops into well-developed dislocation veins, and initial dislocation cells emerge near grain boundaries. The dislocation veins are wide and arranged in parallel along a single slip direction with blurred boundaries, and a large number of free dislocations remain in the gaps, indicating that the substructure is in an unstable evolutionary state. Stress concentration near grain boundaries induces the alternation of single slip and multiple slip, forming cellular substructures with low closure degree and ambiguous boundaries. Due to the relatively low strength and hardness of ferrite phase, the internal deformation is more remarkable, and complete dislocation cell substructures are more likely to develop.

**Figure 22:**
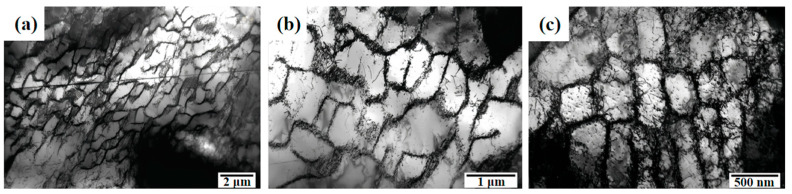
TEM micrographs showing the microstructural evolution of 316L stainless steel at 293 K and a strain amplitude of 0.9% at 530 cycles: (**a**) dislocation morphology, (**b**) dislocation walls, (**c**) magnified view of dislocations.

**Figure 23:**
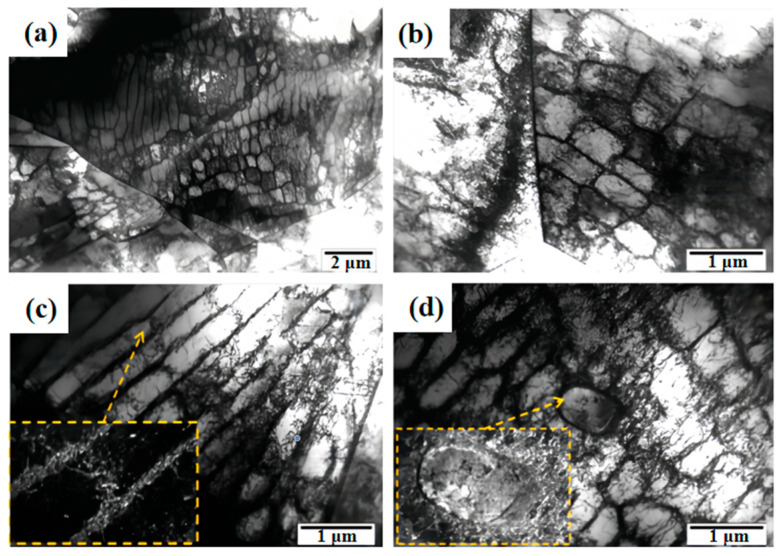
TEM micrographs showing the microstructural evolution of 316L stainless steel at 293 K and a strain amplitude of 0.9% at 837 cycles: (**a**) dislocation morphology, (**b**) dislocation walls, (**c**) magnified view of dislocations, (**d**) ferrite grain.

When loaded to the secondary hardening stage (530 cycles), the microstructural characteristics are displayed in Figure 22a–c. The dislocation substructures are further optimized and dominated by elongated cellular morphologies. At high magnification, dislocation veins become denser with obviously reduced cell wall thickness. The closure degree of dislocation cells increases, and the number of free dislocations inside the cells decreases significantly. New dislocation walls generate inside some cells, realizing secondary segmentation and refinement of microstructures. Although equiaxed dislocation cells sporadically appear in local regions, elongated structures still dominate the whole microstructure. The inhomogeneity of dislocation evolution is closely related to the initial microstructure of base material and the uneven distribution of alloying elements. The TEM micrographs from the room temperature fatigue fracture stage (837 cycles) are shown in Figure 23a–d. The dislocation configuration has fully evolved and is mainly characterized by uniformly distributed equiaxed mature dislocation cells with an overall labyrinth-like morphology. Compared with the secondary hardening stage, the dislocation cells at fracture state are smaller in size and exhibit a more uniform distribution, and no new dislocation walls are generated inside the cells. Partially evolved elongated cellular structures remain in local regions, and dislocation veins extend parallel along the primary slip plane. New dislocation veins perpendicular to the primary slip plane can be observed under dark-field imaging. The ferrite phase still maintains cellular substructures, and its grain boundaries disturb the regular distribution of dislocation cells in the austenite matrix, further aggravating the inhomogeneity of microstructural evolution.

**Figure 24:**
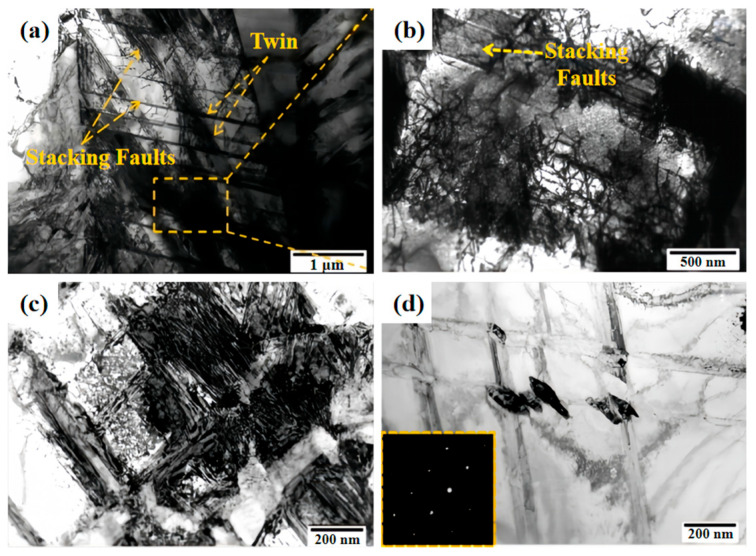
TEM micrographs showing the microstructural evolution of 316L stainless steel at 77 K and a strain amplitude of 0.9% at 50 cycles: (**a**) shear band morphology, (**b**) dislocation distribution, (**c**) matrix regions partitioned by shear bands, (**d**) nucleation sites of martensite.

Unlike the deformation mechanism at 293 K, the coupling effect of low temperature and cyclic strain at 77 K changes the microstructural evolution path, defect configuration, and phase transformation behavior of 316L stainless steel during fatigue, presenting distinct microscopic characteristics. The microstructure at the cyclic hardening stage (50 cycles) is shown in Figure 24a–d. Under the combined action of low temperature and high strain amplitude, a large number of parallel shear bands are generated in the matrix and interwoven into a network structure, accompanied by the development of dense deformation twins and stacking faults. These features serve as the key microscopic distinctions between cryogenic and room temperature fatigue. Dislocations are dominated by high-density entanglement, and regions with high dislocation density can directly induce the nucleation of stacking faults. The dislocation morphology is mainly characterized by accumulation, without distinct and complete dislocation wall structures. The narrow grain regions divided by shear bands restrict the further slip and expansion of dislocations, keeping defects in a high-density accumulation state. The intersection areas of shear bands possess high defect density and concentrated stored energy, providing favorable sites for the preferential nucleation of α′-martensite.

**Figure 25:**
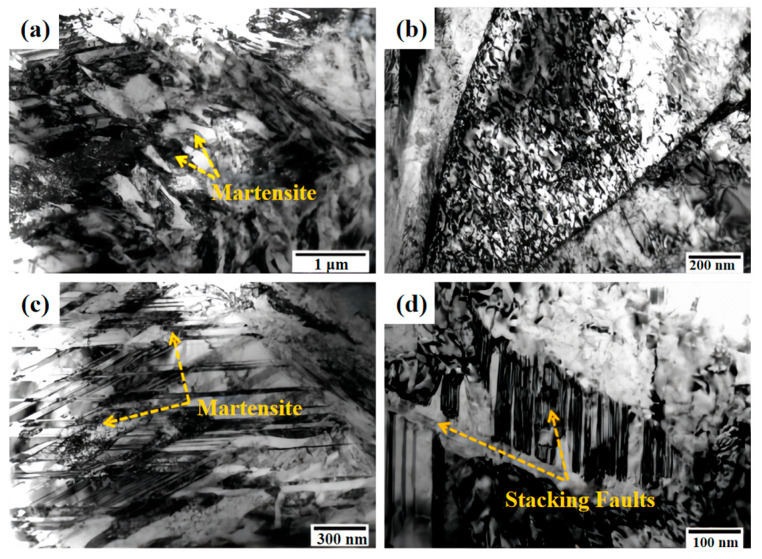
TEM micrographs showing the microstructural evolution of 316L stainless steel at 77 K and a strain amplitude of 0.9% at 1700 cycles: (**a**) distribution of martensite, (**b**) dislocations within martensite, (**c**) dense shear bands, (**d**) high-density stacking faults.

**Figure 26:**
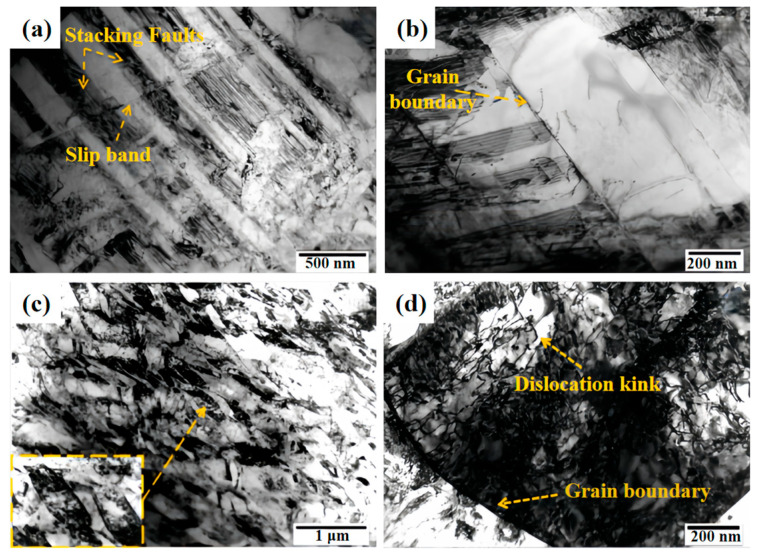
TEM micrographs showing the microstructural evolution of 316L stainless steel at 77 K and a strain amplitude of 0.9% at 4025 cycles: (**a**) stacking faults and slip bands, (**b**) grain boundary morphology, (**c**) distribution of martensite, (**d**) dislocation pile-ups within martensite.

At the cyclic stable stage (1700 cycles), the corresponding microstructure is displayed in Figure 25a–d. The microstructure enters a stable evolutionary state. The supercooling degree at low temperature coupled with cyclic loading provides sufficient driving force for the phase transformation from austenite to martensite, leading to a marked increase in martensite content. Dislocations are mostly distributed inside martensite grains and form high-density pile-ups at grain boundaries. Compared with the cyclic hardening stage, the density and mesh compactness of shear bands are further improved in the stable stage, and mutual penetration and blocking effects occur among shear bands. Stacking fault bands are formed by the continuous accumulation of identical stacking faults along slip planes, and the overall defect configuration tends to be stable. The TEM results at the cryogenic fatigue fracture stage (4025 cycles) are presented in Figure 26a–d. A large number of stacking faults remain in the matrix and can be penetrated by slip bands. Martensite continuously nucleates within and at the intersection of stacking faults. Grain boundaries evidently hinder the propagation of stacking fault bands, while a relatively low dislocation density is maintained inside austenite grains. Deformation twins can extend across grain boundaries and achieve grain refinement. At this stage, the content of α′-martensite is basically consistent with that at the cyclic stable stage, indicating that the phase transformation reaches saturation. Volume expansion and shear distortion induced by phase transformation stabilize the austenite matrix. Dislocation proliferation and motion are concentrated primarily within martensite grains, with obvious pile-up at grain boundaries. The microscopic defect morphology is characterized by a small number of stepped structures and large-scale high-density stacked structures.

At 293 K, fatigue deformation is dominated by the sequential evolution of dislocation slip, dislocation veins, and dislocation cells. Microstructural evolution relies on the activation of slip systems and the reconstruction of dislocation configurations, accompanied by insignificant phase transformation. In contrast, the deformation mechanism at 77 K is synergistically controlled by shear bands, stacking faults, and deformation twins [45]. Low temperature markedly suppresses the long-range slip of dislocations and promotes the early initiation of deformation-induced martensitic transformation, which quickly reaches a saturated state. The significant differences in defect configurations, substructure morphologies, and phase transformation processes between the two temperatures account for the fundamental microscopic mechanism underlying the variations in cyclic hardening and softening response, fatigue life, and fracture mode of 316L stainless steel in cryogenic environments.

**Figure 27:**
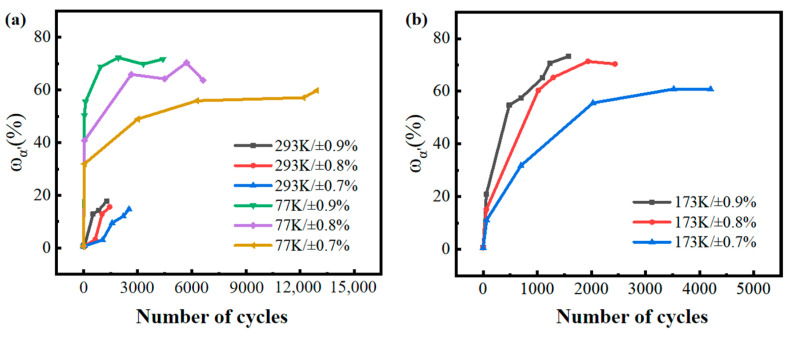
Evolution of α′-martensite mass fraction in 316L stainless steel during cyclic loading. (**a**) Variation of α′-martensite mass fraction with fatigue cycle number at different strain amplitudes at 77 K and 293 K; (**b**) Variation of α′-martensite mass fraction with fatigue cycle number at different strain amplitudes at 173 K.

The influence of temperature on deformation-induced martensitic transformation was quantitatively assessed by analyzing the variation of the α′-martensite mass fraction with the number of fatigue cycles under different conditions, as shown in Figure 27.

Martensite content is governed by the coupling of temperature and deformation level, directly determining the cyclic stress response. At room temperature, the martensite fraction remains very low before the cyclic softening stage, increases rapidly from the cyclic softening stage to half of the fatigue life, and then rises at a slightly slower rate. This evolution corresponds closely to the three-stage cyclic stress response: initial hardening is dominated by dislocation accumulation; cyclic softening results from dislocation annihilation and cross-slip activation, with only a minor contribution from martensitic strengthening; and secondary hardening is driven jointly by the formation of dense dislocation cells and the rapid increase in martensite content. At 77 K and 173 K, the evolution trend of martensite content closely matches the cyclic stress curves: it remains low within the first 10 cycles, corresponding to the initial slow increase in cyclic stress; rises rapidly between 10 and 100 cycles, corresponding to rapid cyclic hardening; and increases more slowly from 100 cycles to half-life, during which stress stabilizes; thereafter, martensite content saturates, and cyclic stress remains steady until fracture. The martensite fraction at low temperatures is significantly higher than that at room temperature, and its contribution to cyclic hardening is far greater.

The fatigue response mechanisms of 316L stainless steel can be systematically elucidated through a comparative analysis of the microstructural evolution and phase transformation behavior under room temperature and deep cryogenic conditions. Stacking fault energy (SFE) is a critical parameter governing the deformation mechanisms of austenitic stainless steel. In the present study, two widely accepted empirical relationships were employed to calculate the SFE values [46,47]. Equation (3) was used to estimate the room temperature SFE based on alloy composition, whereas Equation (4) was adopted to account for the temperature dependence of SFE through a linear correction with absolute temperature.

(3) $$\gamma_{SFE}^{RT}=-53+6.2\left(Ni\right)+0.7\left(Cr\right)+3.2\left(Mn\right)+9.3\left(Mo\right)$$

(4) $$\gamma_{SFE}=\gamma_{SFE}^{RT}+0.05\left(T-293\right)$$

In these equations, Ni, Cr, Mn, and Mo denote the mass fractions of the corresponding alloying elements, while T represents the absolute temperature in Kelvin. Based on these relationships, the SFE values of 316L stainless steel at 293 K and 77 K were calculated to be 45 mJ/m^2^ and 35 mJ/m^2^, respectively, indicating a pronounced reduction in SFE with decreasing temperature.

In the 77 K cryogenic environment, the combination of low stacking fault energy, high strain amplitude, and low temperature promotes dislocation accumulation and local stress concentration, inducing interactions between mechanical twins and stacking faults that form a networked shear band structure, thereby providing abundant effective nucleation sites for martensitic transformation [48]. During the cyclic stabilization stage, the martensite phase and shear bands jointly restrict dislocation motion, and the short-range resistance to dislocation movement increases due to low temperature, resulting in a stabilized cyclic stress response. In the later stages of fatigue, the martensite content reaches saturation, and macroscopic crack initiation and propagation eventually cause a rapid drop in stress levels.

## 5. Conclusions

This study systematically investigated the quasi-static tensile and strain-controlled low-cycle fatigue behavior of 316L austenitic stainless steel at 293 K, 173 K, and 77 K, combined with microstructural characterization to reveal the effects of cryogenic environments on mechanical behavior, cyclic deformation, and fatigue damage mechanisms. Temperature has a significant influence on the static mechanical properties of 316L stainless steel. As temperature decreases, both the yield strength and ultimate tensile strength increase markedly, while ductility slightly decreases but remains sufficient for low-temperature applications. Hardness increases synchronously with strain and low-temperature effects, and cryogenic conditions notably enhance the strain-hardening response. When subjected to low-cycle fatigue, specimens at room temperature exhibit three stages of initial hardening, cyclic softening, and secondary hardening, whereas under low-temperature conditions continuous cyclic hardening rapidly stabilizes. Fatigue life increases significantly with decreasing temperature and strain amplitude, with low temperature showing a particularly pronounced effect on high-strain fatigue life. At the microstructural level, fatigue deformation at room temperature is dominated by the evolution of dislocation cell structures. In cryogenic environments, reduced stacking fault energy induces extensive shear bands, deformation twins, and α′-martensitic transformation. The martensite content rapidly saturates and dominates cyclic hardening behavior. The observed fatigue response is closely associated with the coupled influence of temperature-dependent cyclic stress evolution, plastic deformation accumulation, and deformation-induced martensitic transformation during cyclic loading. Cryogenic conditions result in fewer observable active crack initiation sites on fracture surfaces, reduce crack propagation rates, and produce finer fatigue striations and smoother stable crack growth regions. Meanwhile, the size and depth of dimples in the final fracture zone are reduced, and the material remains dominated by a ductile fracture mode at low temperatures. The results demonstrate that, under cryogenic conditions, fatigue damage is synergistically controlled by dislocation motion, twin formation, and martensitic transformation. These findings provide important experimental data and theoretical support for understanding the cryogenic fatigue behavior and fatigue-resistant design of 316L stainless steel.

## Figures and Tables

**Table 1:** 316L stainless steel chemical composition.

Element	C	Si	Mn	P	S	Cr	Mo	Ni	N
ω (%)	0.027	0.53	1.48	0.035	0.015	16.28	2.11	10.15	0.033

**Table 2:** Fitted parameters of the Manson–Coffin–Basquin model.

Temperature	$\sigma_{f}^{\prime }$ (MPa)	E (MPa)	b	$\varepsilon_{f}^{\prime }$	c
77 K	7423	166,351	−0.1954	0.0103	−0.2471
173 K	3263	148,999	−0.1473	0.1318	−0.5370
293 K	1516	169,499	−0.1632	0.0742	−0.3348

## Data Availability

The original contributions presented in this study are included in the article. Further inquiries can be directed to the corresponding author.
